# A Review of the Research Applications of *Centipeda minima*

**DOI:** 10.3390/molecules29010108

**Published:** 2023-12-23

**Authors:** Jiajun Liu, Wenying Zheng, Yifan He, Wanying Zhang, Zhanhao Luo, Xiaotian Liu, Xingyan Jiang, Fanxin Meng, Liyan Wu

**Affiliations:** School of Pharmacy and Food Science, Zhuhai College of Science and Technology, Zhuhai 519000, China; 13653006033@163.com (J.L.); zwy13590874266@163.com (W.Z.); m18520220978@163.com (Y.H.); zz13632090273@163.com (W.Z.); m18826521183@163.com (Z.L.); 18929737282@163.com (X.L.); 13450117285@163.com (X.J.); mfx@zcst.edu.cn (F.M.)

**Keywords:** *Centipeda minima*, chemical composition, pharmacological activities, mechanisms of action, safety

## Abstract

*Centipeda minima* is a traditional Chinese medicine with wide applications and diverse pharmacological effects. Scholars have conducted extensive studies on its relevant clinical applications, especially its remarkable efficacy in cancer treatment. This paper thoroughly investigates the chemical composition and identification, pharmacological effects, and toxicity, along with the safety of *Centipeda minima*, so as to lay the foundation for corresponding clinical applications and product development. Furthermore, as global scholars have conducted extensive research on such clinical applications and made significant progress, the future development and utilization of *Centipeda minima*’s active ingredients to create novel drugs are of great clinical significance.

## 1. Introduction

*Centipeda minima* [1] is also known as stone caraway, lotus grass, fish dipping grass, chicken gut grass, manchurian and ground pepper, etc., and the scientific name of its original plant is *Centipeda minima* (L.) *A. Braun et Aschers*, which is a common Chinese herb belonging to the genus stone caraway in the family of Apiaceae [2,3]. This soft annual herb has a typical height of 3–20 cm, with head-like and sessile inflorescences measuring 3–4 mm in diameter [4]. First recorded in the *Materia Medica for Dietotherapy*, *Centipeda minima* was subsequently mentioned in the *Compendium of Materia Medica* [5]. Its entire plant can be utilized for medicines, and its harvest time is summer and autumn when blooming. After harvest, the plants should be cleaned and dried. With a spicy flavor and warm feature, *Centipeda minima* belong to the lung meridian in traditional Chinese medicine, or literally, the passages through which vital energy circulates, regulating bodily functions relevant to the lung. This herb has significant effects such as clearing away heat and toxins, promoting diuresis, reducing phlegm retention and removing jaundice, eliminating swelling and pain relief, dissipating wind-cold syndrome, clearing the nostrils, relieving coughs and is primarily for symptoms including wind-cold headaches, coughs and sputum, stuffy noses, and running noses [6]. Specifically, the *Chinese Pharmacopoeia* (2020 edition) has extensive details of *Centipeda minima* [7]. According to the *Chinese Materia Medica*, this herb is effective in expelling wind and clearing orifices, along with detoxifying, and reducing swellings [8]. As per the *Materia Medica Hui Yan*, *Centipeda minima* is a medicinal substance that can unblock the nine passages in the human body, particularly the nasal pathways [9]. Throughout the profound history of traditional Chinese medicine in China, *Centipeda minima* has been applied to treat wind-cold infections and coughs, while also alleviating headaches and nasal congestion. Nowadays, *Centipeda minima* is also used in dietary therapy, commonly in dishes for clearing heat and removing toxins, reducing swelling and relieving pain. It can be noted that there has been a growing interest in the clinical use of this herb due to its diverse pharmacological effects [10] in recent years. Typically, *Centipeda minima* hirsutum extracts (ECMs) have various pharmacological effects [10], such as anti-allergic rhinitis, together with anti-tumor, anti-allergic, anti-inflammatory, and anti-mutagenic features. In addition, it also has good safety and tolerance, which can be demonstrated by the fact that ECM extracts can protect neurons from oxidative stress-induced damage, and the percentage of adverse effects is relatively low during clinical use [11,12]. This paper summarizes the chemical composition, pharmacological activity, and application of *Centipeda minima* in treating related cancers and evaluates the safety and toxicity of *Centipeda minima* so as to provide references for future applications. In summary, as a common Chinese herb, *Centipeda minima* has been widely used in traditional medicine, and it also has the potential for a broad clinical application in modern medicine. Through relevant reviews, this paper can provide a comprehensive theoretical basis and insights for further research and the clinical application of *Centipeda minima*.

## 2. Chemical Composition of *Centipeda minima*

### 2.1. Chemical Composition of Centipeda minima

#### 2.1.1. Volatile Oils

*Centipeda minima* has abundant volatile oils, with a content of about 0.1%. According to the GC-MS analysis of the whole plant [13], the volatile oil compositions in *Centipeda* minima are as follows: eucalyptol, camphor, Verbenol(-)-VERBENONE Transacetic permethrin, Carvol, 1,2,3,6-Tetramethylbicyclo[2.2.2]octa-2,5-diene, Isocaryophyllene, caryophyllene, Bergamotol, 6,6-Dimethyl-2-methylenebicyclo[3.1.1]heptane, Linacol acetate. Among them, Transacetic permethrin has the highest content of 59.06%. In a comparative study between supercritical carbon dioxide extracts and the hydrodistillation of volatile oils from *Centipeda minima* [14], supercritical carbon dioxide extracts mainly contain components with higher molecular weights (number of carbon atoms ≥ 15) and higher boiling points. Among them, Transacetic permethrin had the highest content, while other components with a high content included Palmitic acid thymol and 5-Methyl-2-(1-methylethyl) ester.

#### 2.1.2. Sterols

Various sterol compounds are present in *Centipeda minima*, including Taraxasterol, taraxasterylpalmitate, taraxasteryl acetate, Arnidiol, β-sitosterol, Spinasterol, Stigmasterol, and Stigmasterol glucoside, totaling eight kinds. These sterols are widely distributed in the plant [15]. Detailed information and structures of Sterols in *Centipeda minima* are shown in Table 1.

#### 2.1.3. Flavonoids

*Centipeda minima* has rich flavonoids, mainly quercetin derivatives. In addition to the already mentioned types of quercetin, quercetin-3-O-β-D-glucuronate, quercetin-3,3′-dimethyl ether, quercetin 3-methyl ether, apigenin, and tangerine, there are also many other flavonoids present in this herb. These compounds have substantial bioactive and pharmacological effects that are widely used in nutraceuticals and traditional herbal medicines. It can be noted that the content and composition of flavonoids in *Centipeda minima* may be affected by various factors, such as the growing environment, seasonal changes, and harvest time. Therefore, further research and analyses of these flavonoids will contribute to a better understanding of their nutritional value and medicinal potential. Detailed information and structures of Flavonoids in *Centipeda minima* are shown in Table 2.

#### 2.1.4. Triterpenoids

Triterpenoid compounds in *Centipeda minima* are mainly in the form of pentacyclic triterpenoid saponins and their aglycones. Alongside Ursane-20(30)-en-3β, 16β-21α-triol, and Taraxasterol acetate, numerous other triterpenoids have been detected in *Centipeda minima*, such as xyloxyplugone and its derivatives, which are of significant medicinal value and biological activity in plants [6]. Detailed information and structures of Triterpenoids in *Centipeda minima* are shown in Table 3.

#### 2.1.5. Guaiac Ligolides and Pseudoguaiacolides

In recent years, several components of (pseudo)guaiacolide sesquiterpenes have been successfully isolated, including Brevilin A, Microhelenin C, Minimolide F [6], Arnicolide D [17], Aricolide C [18], 6-O-angeloylenolin (6-OA) [19], Minimolide G, Minimolide H [20], Seneciol dihydropyridyl pyridyl [16], angelica acetic acid glycerin [16], and 2β-light yl-2,3-dihydro-6-0-angelica polystemic cabbage [21]. Detailed information and structures of Guaiacolides in *Centipeda minima* are shown in Table 4.

#### 2.1.6. Other Compounds

Moreover, several thymol derivatives have been discovered in *Centipeda minima*, enriching the variety of its chemical composition [15,22], including 8,10-dihydroxy-9-isobutyroyloxythymol, 8-hydroxy-9,10-diisobutyroyloxythymol, and numerous other thymol derivatives. These compounds have key bioactive functions in plants, such as exhibiting antioxidant and antimicrobial activities. Furthermore, *Centipeda minima* contain several other constituents, including tannins, resins, proteins, epitaxanthinol, and uracil, which enhance its pharmacological activity [15]. Additionally, some organic acids have also been found in *Centipeda minima*, including benzoic acid, palmitic acid, artemisinic acid, pentadecanoic acid, palmitic acid, and octadecanoic acid [23]. As organic acids have diverse biological activities, including antimicrobial, anti-inflammatory, and antioxidant effects in plants, they are widely applied in the pharmaceutical and food sectors. Detailed information and structures of Other compounds in *Centipeda minima* are shown in Table 5.

## 3. Effects of *Centipeda minima* on Cancer/Tumor and Related Mechanisms

### 3.1. Nasopharyngeal Carcinoma NPC (Medical)

According to previous research, CMX and sesquiterpene lactone compounds isolated from *Centipeda minima* exhibit growth inhibitory and apoptosis-inducing effects on nasopharyngeal carcinoma cells (NPCs). In particular, Brevilin A is a sesquiterpene lactone isolated from CM, and its effects on cancer cell lines are applicable to several other cancers, including breast and lung cancers. In addition, Arnicolide D and Arnicolide C are also sesquiterpene lactones found in CM that share the same features as brevilin A. To investigate the potential of Arnicolide D’s anti-nasopharyngeal carcinoma effects and related mechanisms, previous experiments examined the effects of Arnicolide C and Arnicolide D in the NPCs of CNE-1, CNE-2, SUNE-1, HONE1, and C666-1. The results showed that arnicolide D could significantly inhibit the proliferation of NPC, regulate the cell cycle, and induce apoptosis.

The study delved into the molecular mechanisms of cell cycle regulation and the induction of apoptosis. The results suggested that arnicolide D can promote cell cycle arrest in the G2/M phase, activate the PI3K/AKT/mTOR signaling pathway, and induce the mitochondrial apoptosis pathway as part of its mechanism of action. Moreover, the potential anti-nasopharyngeal carcinoma effects of arnicolide D may rely on the interactions among signal pathway network components, indicating its anti-proliferative and apoptosis-inducing activity in NPCs [29].

On the contrary, CMX exhibited antiproliferative properties on NPCs while maintaining low toxicity to normal cells. Research on this aspect has been conducted where various concentrations of CMX have been applied to CNE-1 NPC cells, and the MTT assay has been used to detect cytotoxicity. Fluorescence microscopy was employed to observe morphological changes in the cells, and flow cytometry was used to evaluate the cell cycle status. Apoptosis and associated protein levels were determined using annexin V-FITC/PI staining and Western blotting. The results demonstrated that CMX (15-50 μg/mL) inhibited the growth of CNE-1 cells in a dose and time-dependent approach and led to cell apoptosis. In particular, toxicity to normal cells (LO2) was relatively minimal. CNE-1 cells were in the G2/M phase when treated with 15, 25, and 40 μg/mL CMX. Furthermore, CMX reduced the Bcl-2 expression, increased Bax expression, and activated caspase-3, caspase-8, caspase-9, and PARP, indicating that its effect on cancer cell lines can be realized through regulating apoptosis-related proteins.

At the molecular level, the treatment of 40 μg/mL of CMX inhibited the activation of the PI3K-AKT-mTOR signaling pathway, demonstrating its crucial role in suppressing NPC proliferation [30]. Furthermore, the supercritical fluid-extracted volatile oils of *Centipeda minima* were found to induce apoptosis in CNX cells by regulating the expression of Bcl-2 family proteins. This process results in dysfunctional mitochondria, promoting the release of cytochrome c into the cytoplasm, which subsequently activates caspase-9. Finally, caspase-3 and caspase-7 were cleaved, culminating in CNX cell death [31]. In summary, various CMXs show anti-nasopharyngeal cancer properties.

### 3.2. Non-Small Cell Lung Cancer

NSCLC is a prevalent malignancy, accounting for 80% of all lung cancer diagnoses, so research into new forms of treatment for the prevention and management of NSCLC is crucial. Recent studies have shown that the ECM, the monomer compound arnica lactone C (ArnC), and DNA cross-linking agents together demonstrate promising effects on the cancer cell lines of NSCLC. More specifically, CMX has anti-inflammatory, anti-tumor, anti-angiogenic, anti-mutagenic, and anti-proliferative pharmacological features, so its ethanol extracts can prevent cancer by inhibiting growth and inducing the apoptosis of tumor cells. Additionally, ArnC is a natural alkaloid with antitumor, antioxidant, and other beneficial pharmacological activities; thus, it can prevent cancer by restricting tumor cell proliferation and encouraging cell death. When used in combination with DNA cross-linking agents, both have the potential to enhance therapeutic effects on NSCLC synergistically, which is a promising therapy for further studies [32].

In the study of adjuvant treatment of NSCLC with *Centipeda minima*, concentrations were used to reduce cell viability to less than 50% so as to test the chemosensitizing effects of ECM, while treatment with relatively low concentrations of cisplatin (CDDP) also caused moderate cytotoxicity to NSCLC cells. As a result, the combination of ECM and CDDP therapy gained a synergistic effect in reducing cell viability in NSCLC; the co-treatment of ECM and mitomycin C (MMC) also produced synergistic cytotoxic effects on A549 and H1299 cells, and the results indicated that ECM was able to sensitize DNA cross-linker-induced cytotoxicity. Here, to assess the safety of the combined treatment strategy, the side effects were further tested when ECM and CDDP were combined to treat mice. The results showed that ECM treatment alone did not result in significant weight loss, hepatotoxicity, or nephrotoxicity, with no significant side effects on the liver or kidneys due to relatively low concentrations of CDDP. In addition, the combination of ECM and CDDP showed no significant toxicity. In brief, ECM is safe for in vivo administration [33].

The ethanolic extract of the ECM of *Centipeda minima* effectively sensitized NSCLC cells to DNA cross-linking agents, leading to apoptosis. Moreover, the ECM inhibited the activation of the Fanconi anemia (FA) pathway and cell cycle checkpoints induced by DNA cross-linkers, thereby significantly increasing DNA damage [34]. In conclusion, the combination therapy of ECM and CDDP showed synergistic positive effects on the cancer cell lines of NSCLC. Furthermore, the findings demonstrate that the ethanol extracts of *Centipeda minima* exhibited great sensitivity to DNA cross-linking agents in NSCLC cells by activating FA [35] and Chk1 signaling, which could potentially be suppressed.

### 3.3. Triple-Negative Breast Cancer

Studies have found that CMX can treat not only rhinitis and sinusitis but also breast cancer. Breast cancer is common among women, and triple-negative breast cancer (TNBC) is the most aggressive subtype with the highest recurrence rate and lowest survival rate. Particularly, TNBC accounts for 5–25% of all breast cancers and is a clinical estrogen receptor (ER), progesterone receptor (PR), and human epidermal growth factor receptor 2 negative tumors. In the experiments exploring the effects of CMX on MDA-MB-231 and MCF-7 human breast cancer cells, CMX’s effects on MDA-MB-231 TNBC breast cancer cells were mainly manifested by the induction of apoptosis, the inhibition of the activity of MMP-9, and obstruction of the cell migration process in breast cancer cells. CMX was found to enhance endogenous and exogenous apoptotic pathways in the MDA-MB-231 TNBC breast cancer cell line and limit the uncontrolled proliferation of cancer cells. Usually, MMP-9 is closely associated with cell migration and invasion in the cancer process, but CMX can curb the invasive development of breast cancer by inhibiting the activity of MMP-9 and blocking the cell migration process. According to in-depth studies, CMX’s mechanism of action should function by inhibiting the potential therapeutic target epidermal growth factor receptor (EGFR) and down-regulating the PI3K/AKT/mTOR, NF-κB, and STAT3 signaling pathways. These signaling pathways play key roles in breast cancer development and are especially closely related to the expression and activity of MMP-9. Overall, CMX has significant effects on breast cancer cells without toxic reactions in the experiments, which could be great assets for the future research and development of TNBC treatment [36].

### 3.4. Liver Cancer

In recent years, research has shown the potential anti-liver cancer properties of *Centipeda minima*. In this aspect, sesquiterpene lactones were extracted from this plant using 95% ethanol before being purified, and their structures were identified using wave spectroscopy. Their ability to inhibit tumor cell proliferation was then tested using the tetramethyl azole blue (MTT) method [17]. In conclusion, the five compounds from *Centipeda minima*, namely brevilin A, microhelenin C, minimolide F, arnicolide C, and arnicolide D, which have been identified to restrain the growth of HepG2 (hepatocellular carcinoma) by obstructing numerous signaling pathways [17,37], such as SCF E3 ligase, STAT3, NF-κB, and PI3K/Akt [38].

### 3.5. Stomach Cancer

Stomach cancer is a global health concern, ranking fifth among the most common cancers and third in cancer-related deaths. Typically, helicobacter pylori infection, age, high salt intake, alongside diets lacking in fruits and vegetables are major risks of developing such a disease. The bacterium destroys the stomach’s lining, leading to chronic inflammation and, ultimately, greater chances of gastric cancer [39]. Furthermore, the gastric mucosa typically experiences years of precancerous changes before final gastric malignancy [40]. Despite a substantial decrease in new cases of gastric cancer worldwide due to lower Helicobacter pylori, the East Asian region still has a high rate of such disease. Additionally, as there are currently no effective Western medications for gastric cancer, new treatments are surely necessary [40].

Today, Chinese medicine is gaining more popularity and acceptance worldwide due to its distinct benefits in cancer treatment, including multiple targets, minimal side effects, and superior therapeutic effects [41]. To be noted, compounds that inhibit cancer metastasis using molecular mechanisms found in Chinese herbal formulas, proprietary Chinese medicines, single Chinese medicines, and Chinese medicine monomers are emerging fields in cancer treatment. In this aspect, traditional Chinese medicine (TCM) plays a unique role in this new field and can inspire novel concepts and approaches accordingly [41].

**Figure 1:**
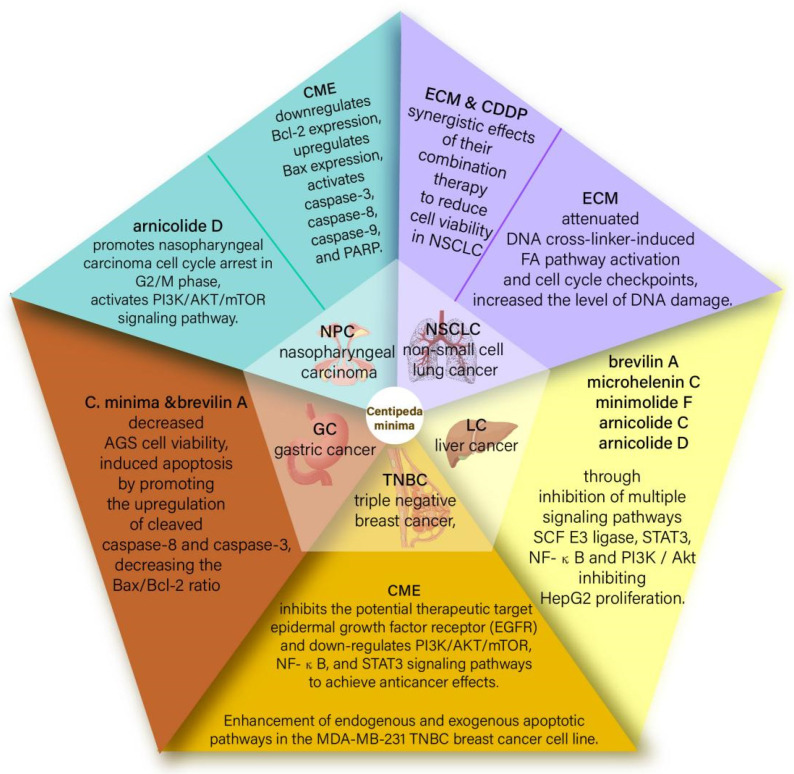
Summary diagram of the mechanism of action of *Centipeda minima* on various cancers.

*Centipeda minima* is an annual herb indigenous to the tropics, particularly in eastern tropical Asia [42]. According to phytochemical investigations, the active components of CMXs and sesquiterpene lactones exhibit marvelous effects on cancer cell lines and chemosensitizing. In particular, Brevilin A, a natural sesquiterpene lactone drug derived from *Centipeda minima,* shows such a property on cancer cells [43]. The main effect of *Centipeda minima* on cancer cell lines is the targeting of the Skp1-Cullin1-F-box protein (SCF) E3 ubiquitin ligase and transcription signal transducer and activator of transcription 3 (STAT3) [38]. Recent studies have confirmed that CCND1, CDK4, and BCL2L1 are the key genes related to anticancer activity and targeted by brevilin A, whereas signaling pathways such as PI3K-Akt-mTOR, JAK-STAT, and MAPK may be part of Brevilin A’s activity on cancer cells. Additionally, both *Centipeda minima* and Brevilin A can reduce AGS cell viability and trigger apoptosis by increasing the expression of cleaved caspase-8 and cleaved caspase-3 while also reducing the Bax/Bcl-2 ratio, with an IC50 of 9.73 1.29 μg/mL for CMX after 24 h. In summary, *Centipeda minima* and Brevilin A are potential herbal medicines for treating gastric cancer [44].Detailed information and structures of the mechanism of action of *Centipeda minima* on various cancers are shown in Figure 1.

## 4. Other Pharmacological Effects of *Centipeda minima*

### 4.1. Anti-Inflammatory and Antioxidant Effects

*Centipeda minima* contain several active components, including polyphenols, flavonoids, triterpenoids, and steroids, which have substantial anti-inflammatory and antioxidant effects. Relevant studies have demonstrated that *Centipeda minima* can eradicate free radicals, decrease oxidative destruction, and protect cells against oxidative damage. Moreover, *Centipeda minima* can decelerate the aging process and ameliorate the body’s antioxidant potential [45]. Its anti-inflammatory effects function by inhibiting the release of inflammatory mediators and reducing the inflammatory response [46]. Furthermore, it can promote lymphocyte activation and proliferation, which enhances the body’s immunity and anti-inflammatory ability [46]. According to a study on the therapeutic role of *Centipeda minima* in psoriasis [11] using an in vitro model of the disease, the effect of the *Centipeda minima* extract (CMX) on macrophages and keratin-forming cells treated with LPS, IL-6, or IFN-y was assessed. More specifically, psoriasis is typified by heightened keratin-forming cell proliferation and immune cell infiltration. The JAK/STAT3 and JAK/STAT1 signaling pathways are crucial in psoriasis development via the stimulation of IL-6 and IFN-y, which are produced by dendritic cells and T lymphocytes. Therefore, blocking JAK/STAT signaling can potentially help psoriasis treatment. Research has also revealed that CMX can reduce pro-inflammatory cytokines by hindering the lipopolysaccharide (PS)-induced phosphorylation of JAK12 and STAT1/3 in macrophages [47]. In addition, CMX reduced chemokine expression and cell proliferation in comparison to rh-IL-6 and rh-IFN-V-induced HaCaT cells, regulating JAK/STAT-mediated inflammatory responses in macrophages and keratinocytes. That is, CMX can also be a treatment for psoriasis [29].

In light of related pharmacological studies [45], the volatile oil of *Centipeda minima* showed not only obvious inhibitory effects in the experiment of cotton ball granuloma in mice but also clear inhibitory effects in the experiment of foot-plantar swelling induced by egg whites in rats. It indicates that such a volatile oil has a certain antagonistic effect on both acute and chronic inflammation by inhibiting the release of the traditional inflammatory mediators histamine and 5-hydroxytryptamine.

Through a model of allergic rhinitis with positive and medicated control groups, along with volatile oil-treated and negative control groups, the pragmatic effect of *Centipeda minima*’s volatile oil on allergic rhinitis was proven [48]. Additionally, during the treatment of allergic rhinitis in guinea pigs, the volatile oil of *Centipeda minima* demonstrated a significant inhibitory effect on eosinophil and mast cell production [49]. The nasal mucosal epithelial tissue of the positive control group with allergic rhinitis exhibited congestion and edema, along with the infiltration of inflammatory cells. Moreover, a substantial number of neutrophils, eosinophils, lymphocytes, and mast cells were noted. Notwithstanding this, the treatment group added with *Centipeda minima*’s volatile oil showed the remarkable mitigation of these aforementioned changes, similar to the negative control group. Finally, pathological changes in the nasal mucosal tissue were reduced, and there was no harm to the nasal mucosa after long-term treatment.

In conclusion, CMX’s anti-inflammatory properties, together with the volatile oil of *Centipeda minima*, are promising treatments for allergic rhinitis. Thus, further research on this aspect can provide novel options for treating respiratory conditions.

### 4.2. Asthma Calming Effect

Sterols, guaiacolactones, monoterpenes, triterpenes and triterpenes saponins, volatile oils, flavonoids, and thymol derivatives are present in *Centipeda minima*, with chrysene acetate as the major volatile oil component. Experiments utilized the guinea pig pharmacological asthma-inducing pharmacodynamics method, and the extraction and separation of the volatile oil of *Centipeda minima* were found to be significant in alleviating asthma [50]. The antitussive effect of such volatile oil on the spasm of tracheal smooth muscle that has been induced by histamine phosphate was ascertained through the gastric-inducing test of guinea pig spray and the contraction of spiral strips of isolated tracheal smooth muscle. The results demonstrate that *Centipeda minima* possess a distinctive antispasmodic effect.

### 4.3. Antitumor Effect

Various active components within *Centipeda minima* have positive effects on fighting against cancer cells. The aforementioned plant species possess inhibitory effects against tumorigenesis together with cancer cell proliferation and metastasis. Furthermore, *Centipeda minima* can facilitate the immunological response and enhance the body’s ability to recognize and eliminate neoplastic cells. Additionally, *Centipeda minima* can also be applied to the prevention and alleviation of various malignant tumors. Sesquiterpene lactone compounds extracted from *Centipeda minima* have inhibitory growth and apoptosis-inducing effects on nasopharyngeal carcinoma cells. While classified as pseudo guaiacolides or guaiacolides, the sesquiterpene lactones in *Centipeda minima* display notable structural differences from those of other origins. The compound’s C/B ring is joined at positions 7 and 8, whereas α- and β-unsaturated lactone groups are present in the A ring. Furthermore, substitutions of methylcrotonyl, angelicinyl, and methacryloyl groups at the six-position contribute to its highly specific structure and confer potential antitumor properties. Specifically, this compound is proven to be pioneering in treating nasopharyngeal carcinoma [30,51]. The α- and β-unsaturated lactone structures of *Centipeda minima* are Michael receptors, enhancing the physiological activity of this compound. Additionally, this plant also exhibits significant inhibitory effects on other tumors, such as colon and lung ones [29,45,52,53].

Relevant studies have shown that the extract (CME) of *Centipeda minima* is positive in dealing with human nasopharyngeal cancer cell lines [29]. Two specific sesquiterpene lactones, namely scopoletin C and scopoletin D, were identified from *Centipeda minima*. Through a detailed investigation into the molecular mechanisms of regulating the cell cycle and inducing apoptosis, scopoletin D inhibits the expression of the cell cycle proteins D3, cdc2, and p. Meanwhile, the expression levels of cell cycle protein B1, cdk6, and Bc1-2 were found to vary with both time and dosage in response to scopolamine D. Furthermore, Sanguinarine D was observed to activate the cystatin protease Conf pathway in the cell cycle and suppress the PI3K/AKT/mTOR and STAT3 signaling pathways. According to this study, STAT3 levels in nasopharyngeal carcinoma tissues are higher than those in normal tissues and negatively correlated with survival rates [54]. Furthermore, compound calendula A has been verified to reduce p-STAT3 expression in various cancers, such as lung, breast, and liver types [55,56]. Scopolamine D suppresses p-STAT3 expression in CNE-2 cells, and this suppression can aid in triggering apoptosis and restraining migration in cancer cells [49,56]. The inhibition of STAT3 activation may further inhibit AKT activation, suggesting the multifactorial nature of the efficacy of scopolamine D in inhibiting cancer cell lines. It was reported that the utilization of the extracellular matrix and monomeric compound ArnC combined with the DNA cross-linking agent could yield a targeted effect on non-small cell lung carcinoma [32]. To explain this, CMX has anti-inflammatory properties, anti-angiogenic, and anti-mutagenic pharmacological effects so as to treat cancer cell lines. Additionally, its extracellular matrix (ECM) can inhibit tumor cell proliferation and induce tumor cell apoptosis. Moreover, ArnC is a natural alkaloid with several pharmacological activities, such as antitumor and antioxidant properties, which can also inhibit tumor cell proliferation and induce tumor cell apoptosis. The combination of both compounds with DNA cross-linking agents can potentially enhance synergistic therapeutic efficiency on non-small cell lung cancer (NSCLC); therefore, further relevant explorations are highly encouraged.

During research into the adjuvant treatment of NSCLC with *Centipeda minima*, the chemosensitizing effect of ECM was tested by concentrations that reduced cell viability to less than 50%. Additionally, even relatively low concentrations of CDDP can also induce moderate cytotoxicity against NSCLC cells. The results revealed that a combination of ECM and CDDP treatment yielded synergistic effects in reducing cell viability in NSCLC while administering ECM and MMC simultaneously led to synergistic cytotoxic effects on both A549 and H1299 cells. Furthermore, CM facilitated the sensitization of DNA cross-linking agent-induced cytotoxicity. In addition, researchers further evaluated the safety of the combined treatment approach by testing its adverse effects on mice. As a result, ECM treatment did not result in significant weight loss, hepatotoxicity, or nephrotoxicity. The low concentration of CDDP used only marginally reduced body weight, with no adverse side effects on the liver and kidneys. Moreover, the combined treatment of ECM and CDDP demonstrated no significant toxicity. In conclusion, ECM is safe to administer in vivo [33].

In summary, ECM enhances the sensitivity of NSCLC cells to DNA cross-linking agents and triggers apoptosis. Mechanistically, ECM decreased the FA (Fanconi anemia) pathway activation and cell cycle checkpoints initiated by DNA cross-linking agents, thus significantly increasing DNA damage [34]. The combination of the ECM and CDDP (cisplatin) treatment demonstrated a synergistic anticancer effect on NSCLC. That is, ECM sensitizes NSCLC cells towards DNA cross-linking agents through the inhibition of FA [35] and Chk1 signaling. This combination therapy is a potential golden treatment strategy against cancer. Detailed information and structures of Utilities of *Centipeda minima* are shown in Table 6.

## 5. Safety and Toxicity of Drug *Centipeda minima*

### 5.1. Safety Evaluation of Centipeda minima

Internally taken, *Centipeda minima* primarily affects the gastrointestinal system [57]. More specifically, *Centipeda minima* produces burning feelings in the pharynx, esophagus, and stomach, plus nausea, vomiting, stomach pain, and even abdominal pain (severe pain may be felt in severe cases) for a period of time after drug consumption [58]. In addition, some patients experience back and whole-body pains. However, relevant clinical uses witnessed few cases of adverse reactions, which indicates that *Centipeda minima* is a relatively safe, mild, and effective herbal remedy. Corresponding studies [59] show that out of 97 patients diagnosed with allergic rhinitis, 19 of them, or 19.55% experienced transient adverse reactions. Furthermore, of these patients, nine individuals, or 9.19% encountered sustained adverse reactions, leading to the discontinuation of the use of *Centipeda minima*. The remaining patients did not report any adverse reactions.

In brief, *Centipeda minima* has wide applications in Chinese medicinal practice as an herbal remedy. Despite some potential adverse reactions, it is generally rated as a relatively risk-free, gentle, and potent herb with significant medicinal values that is extensively employed in TCM practice. Accordingly, to pursue the higher efficiency and safety of *Centipeda minima*, it is advisable to seek guidance from a medical expert or professional Chinese medicine practitioner.

### 5.2. Toxicity Evaluation of Centipeda minima

According to professional studies [11], a neurodegenerative mouse model was adopted to showcase CMX’s effect on protecting neurons from oxidative stress-induced damage. The results suggest that CMX may be a safe option for in vivo treatment. In order to further assess its toxicity, a series of examinations were then conducted, with special attention given to the effects of CMX on the liver and kidneys. As a result, CMX did not have any significant toxic reaction in these organs, thereby further supporting the safety of CMX [11].

To be noted, although with the preliminary evidence of CMX’s safety, results are based solely on animal experiment outcomes. Previously, there were limited studies on the potential organ-specific toxicity of *Centipeda minima* and insufficient scientific data to assess its potential effects on target organs. To better evaluate the safety and possible side effects of CMX, additional studies such as toxicity studies and clinical trials are necessary. Therefore, it is not fully evident that *Centipeda minima* has low toxicity without further investigations. However, in the above-mentioned findings against a neurodegenerative mouse model, ECM may be relatively safe for in vivo treatment [12], which provides a theoretical basis and a research direction for the potential toxicity of *Centipeda minima* in humans.

## 6. Conclusions

*Centipeda minima* refers to the whole plant of *Centipeda minima* (L.) *A. Braun et Aschers*, which is a traditional Chinese medicinal material utilized for treating wind-cold syndrome, wind pain, coughs, phlegm, nasal congestion, and runny nose. It has a profound history and wide applications, making it a promising subject of study.

The primary bioactive constituents of *Centipeda minima* are volatile oils, terpenes, flavonoids, and organic acids, all exhibiting extensive pharmacological effects. According to the compounds studied, volatile oil has demonstrated properties of relieving allergic rhinitis, anti-inflammatory responses, and dealing with cancer cell lines, with its ethanol extracts showing antimicrobial activity. Furthermore, its sesquiterpene lactone compounds are found to have a certain inhibitory effect on cancer cell proliferation, finally contributing to tumor treatment efficiency. In addition, *Centipeda minima* decoction is effective in providing hepatoprotection and eliminating drug-resistant plasmids. All these findings support the potential health benefits of *Centipeda minima* compounds [6,10].

Based on the pharmacological properties of *Centipeda minima*, a study was conducted to explore its clinical applications. Accordingly, *Centipeda minima* was remarkable in numerous cases, including rhinitis, whooping cough, headaches, lithiasis, surgery diseases, facial paralysis, hookworm tail col infection, and malaria. More specifically, in treating rhinitis, *Centipeda minima* can effectively manage acute, chronic, and allergic forms. In terms of pertussis treatment, fresh *Centipeda minima* syrup shows a notable effect on healing pertussis in children, demonstrating quickness and a high cure rate. Regarding lithiasis, the combination of *Centipeda minima* with Ugin decoction and oral administration is a favorable solution. In addition, combining *Centipeda minima* with Western medicine is a remarkable therapy for migraines. In terms of surgical treatment, *Centipeda minima* has been successful in external applications to treat soft tissue contusions, acute lumbar sprains, lumbar muscle strain, joint injuries, rheumatic pain, etc. As for facial paralysis, *Centipeda minima* used externally, alone or in combination with anti-cancer drugs has shown positive outcomes. To treat hookworm tail col infection, a *Centipeda minima* puree is made by mixing it with saliva and applying it externally on the infected area. Additionally, in dealing with malaria, an injection of *Centipeda minima* is administered before disease attacks, resulting in high therapeutic response rates and significant efficacy [10]. Therefore, it is evident that *Centipeda minima* has significant potential for clinical treatment and further research.

In terms of safety, researchers have verified that *Centipeda minima* poses no notable irritation to the nasal mucosa and major organs during in vivo use, so it is considered safe for medicinal purposes [57].

For the production of *Centipeda minima* dosages, this herb is commonly processed into a powder or drops for clinical use. Additionally, it is often combined with other medical ingredients for sprays that are predominantly employed in treating rhinitis.

In summary, *Centipeda minima* is a mild and non-irritating Chinese herbal medicine with a wide range of medicinal effects in clinical practice. It displays many characteristics in pharmacology and has good prospects for development and utilization. Notably, despite in-depth experimental studies on rhinitis [12], many other aspects of this herbal medicine remain to be further studied. Therefore, future pharmacological research on *Centipeda minima* can be combined with modern medical technology so as to generate novel dosage forms and drugs or further explore its medicinal properties. Furthermore, the scientific development and application of *Centipeda minima* resources in China can also alleviate the shortage of some TCM resources while also fostering and enhancing TCM.

## Figures and Tables

**Table 1:** Sterols in *Centipeda minima*.

Chemical Substances	Molecular Formula	Molecular Weight	CAS	Chemical Structure Formula	Brief Description of the Role	Reference
Taraxasterol	C_30_H_50_O	426.72	127-22-0	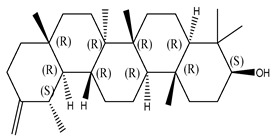	It is separated from Taraxacum and has anti-inflammatory and positive therapeutic effects on cancer cell lines.	[15]
Taraxasteryl palmitate	C_46_H_80_O_2_	665.13	29803-90-5	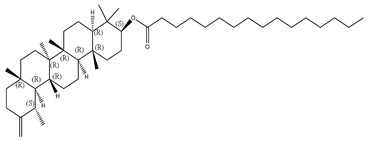	Antioxidant, anti-inflammatory, anti-tumor, lipid-lowering, and visual protection effects.	[15]
Taraxerol acetate	C_32_H_52_O_6_	468.76	2189-80-2	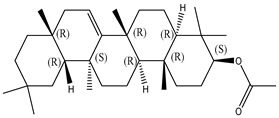	Positive therapeutic effects on cancer cell lines and the induction of cell apoptosis.	[15]
Arnidiol	C_30_H_50_O_2_	442.72	6750-30-7	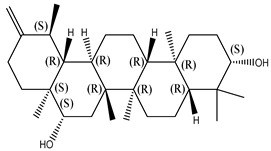	It has a certain anti-inflammatory effect. It can quickly reduce swelling and clear congestion, promote healing, and has the ability to protect the body from bacterial infections, with certain anti-inflammatory and antiviral effects.	[15]
β-sitosterol	C_29_H_50_O	414.71	145428-11-1	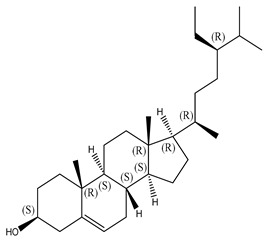	Reduce serum cholesterol.	[15]
Spinasterol	C_29_H_48_O	412.69	481-18-5	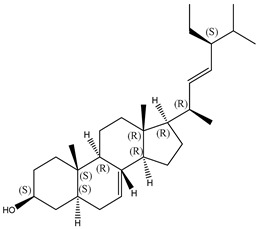	It is widely present in various plants, and it is an important component of plant cells	[15]
Stigmasterol	C_29_H_48_O	412.69	83-48-7	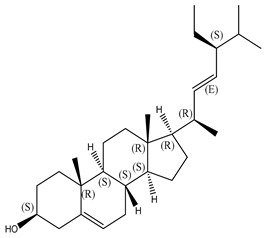	It can reduce the activity of cholesterol and has positive therapeutic effects on cancer cell lines, antipyretic, anti-inflammatory, and immunomodulatory effects.	[15]
Stigmasterol glucoside	C_35_H_58_O_6_	574.84	19716-26-8	/	The prevention and treatment of cardiovascular diseases such as hypertension and coronary heart disease.	[15]

**Table 2:** Flavonoids in *Centipeda minima*.

Chemical Substances	Molecular Formula	Molecular Weight	CAS	Chemical Structure Formula	Brief Description of the Role	Reference
Quercetin	C_15_H_10_O_7_	302.2	117-39-5	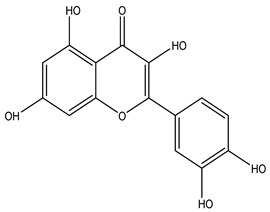	Antioxidant, anti-inflammatory, cough-relieving, and asthma-relieving, heat-clearing and detoxifying, enhancing immunity, and preventing allergies.	[15]
Apigenin	C_15_H_10_O_5_	270.2	520-36-5	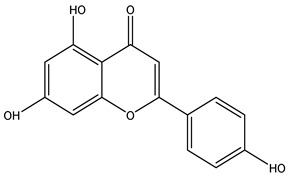	Antioxidant, anti-inflammatory, anti-tumor, lipid-lowering, and visual protection effects.	[15]
Nobiletin	C_21_H_22_O_8_	402.3	478-01-3	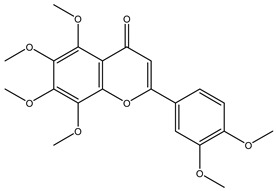	Anti-blood cell agglutination, anti-thrombosis, anti-inflammatory, antiviral, anti mutation, anti-allergic.	[15]
Quercetin -3-O-β-D-glucuronide	C_21_H_20_O_12_	464.4	482-36-0	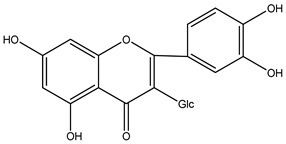	Antioxidant and antiatherosclerotic effects.	[16]
Quercetin-3,3′-dimethyl ether	C_17_H_14_O_7_	330.3	18085-97-7	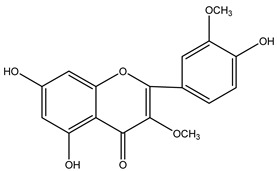	Antioxidant activity.	[16]
Quercetin-3-methyl ether	C_16_H_12_O_7_	316.3	480-19-3	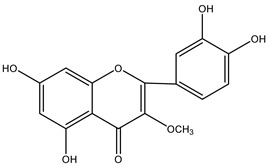	Inhibiting proliferation and inducing the apoptosis of colorectal cancer cells.	[16]

**Table 3:** Triterpenoids in *Centipeda minima*.

Chemical Substances	Molecular Formula	Molecular Weight	CAS	Chemical Structure Formula	Brief Description of the Role	Reference
Ursane-20(30)-en-3β,16β-21α-triol	C_30_H_50_O_3_	458.7	4547-28-8	/	Has antibacterial activity.	[16]
Taraxasterol acetate	C_31_H_52_O_2_	456.7	6426-43-3	/	Anti-hepatitis.	[16]
Friedelin	C_30_H_50_O	426.7	559-74-0	/	Anti inflammatory, antioxidant, and has positive therapeutic effects on cancer cell lines.	[16]

**Table 4:** Guaiacolides in *Centipeda minima*.

Chemical Substances	Molecular Formula	Molecular Weight	CAS	Chemical Structure Formula	Brief Description of the Role
Brevilin A	C_20_H_26_O_5_	346.42	16503-32-5	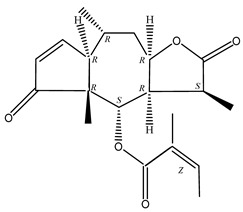	Anti-cancer
Microhelenin C	C_20_H_26_O_5_	346.42	63569-07-3	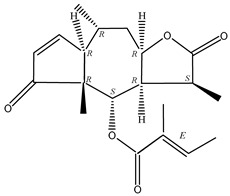	Anti-cancer
Arnicolide D	C_19_H_24_O_5_	332.39	34532-68-8	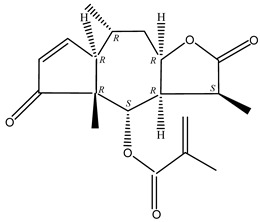	Anti-cancer
Arnicolide C	C_19_H_26_O_5_	334.39	34532-67-7	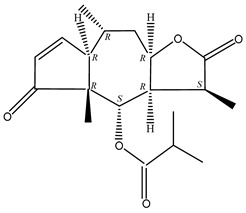	Anti nasopharyngeal carcinoma
Minimolide G	C_20_H_13_O_6_	337.42	/	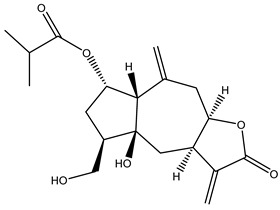	Anti nasopharyngeal carcinoma
Minimolide H	C_20_H_13_O_6_	337.42	/	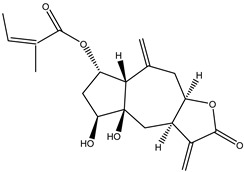	Anti nasopharyngeal carcinoma
Forilenalin	C_15_H_20_O_4_	264.32	54964-49-7	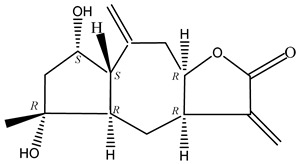	Anti-tumor
2β-Light-2,3-dihydro-6-O-angenoylpolygonum chrysin	C_20_H_28_O_6_	364.32	/	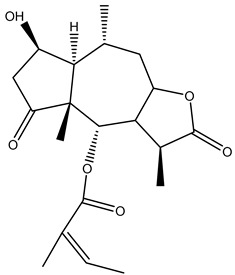	Anti nasopharyngeal carcinoma
Minimolide F	C_19_H_26_O_5_	334.40	1367351-41-4	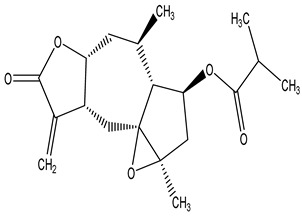	Anti-cancer
6-O-angeloylenolin	C_20_HO_5_	/	/	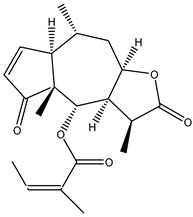	/
Senecioylplenolin	C_22_HO_5_	/	/	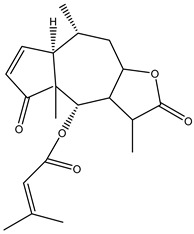	/
Angelicacid florilenalin	C_20_HO_3_	/	/	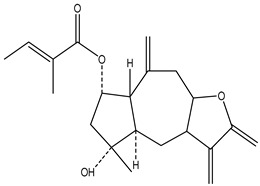	/

**Table 5:** Other compounds in *Centipeda minima*.

Chemical Substances	Molecular Formula	Molecular Weight	CAS	Chemical Structure Formula	Brief Description of the Role	Reference
Thymol	C_10_H_14_O	150.218	89-83-8	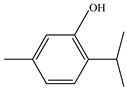	Mainly from the genus Thymus, it can promote the movement of tracheal cilia, favoring the secretion of tracheal mucus and acting as an expectorant.	[24]
Cornusiin	C_68_H_59_O_44_	1571.1	95263-69-7	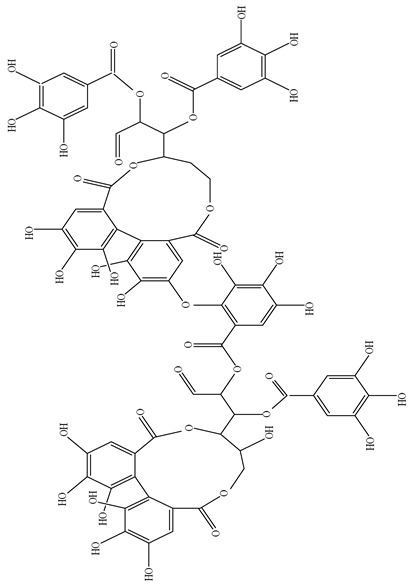	Anti-oxidative stress protects nerve cells and prevents AD (Alzheimer’s disease).	[25]
Uracil	C_4_H_4_N_2_O_2_	112.087	66-22-8	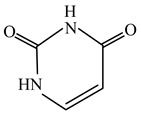	/	/
Benzoic acid	C_7_H_6_O_2_	122.12	65-85-0	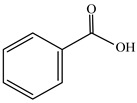	Has an anti-endotoxin effect.	[26]
Palmitic acid-13C	C_1513_CH_32_O_2_	257.417	287-100-87-2	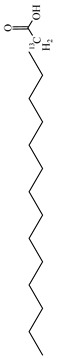	Excess palmitic acid causes apoptosis in some cells.	[23]
Artemisinic acid	C_15_H_22_O_2_	234.334	80286-58-4	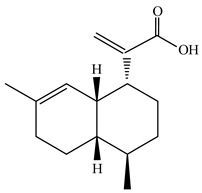	Artemisinic acid is an important intermediate in the biosynthetic pathway of the antimalarial drug artemisinin, which itself has some antimalarial effects.	[27]
Pentadecanoic acid	C_15_H_30_O_2_	242.398	1002-84-2	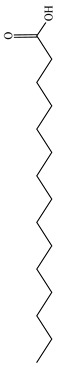	Pentadecanoic acid reduces the total serum cholesterol, inhibits inflammatory factors, and prevents the destructive effects of diabetes on pancreatic islet cells.	[28]
cis-Vaccenic acid	C_18_H_34_O_2_	282.461	506-17-2	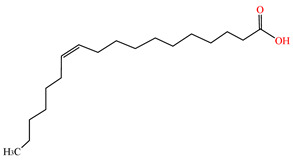	/	/

**Table 6:** Utilities of *Centipeda minima*.

Pharmacological Activities	Functional Roles	References
antioxidant	Contains a variety of active ingredients such as polyphenols, flavonoids, triterpenes, steroids, etc. Ability to scavenge free radicals and reduce oxidative damage Slow down the aging process	[\]
anti-inflammatory (medicine)	Inhibits the release of inflammatory mediators Promotes the activation and proliferation of lymphocytes	[11,29,45,46,47,48,49]
asthma	Antispasmodic effect on histamine the phosphate-induced spasm of the tracheal smooth muscle	[50]
antitumor effect	The inhibition of tumor cell growth and spread The ability to scavenge free radicals, reduce oxidative damage and protect cells from oxidative damage. The prevention and treatment of many tumor diseases Regulating cell cycle proteins and multiple signaling pathways Enhancing the sensitivity of DNA cross-linking agents	[29,30,32,33,34,51,52,53,54,55,56]
